# Current Insights into the Potential Role of fMRI in Discovering the Mechanisms Underlying Obesity

**DOI:** 10.3390/jcm12134379

**Published:** 2023-06-29

**Authors:** Hanna Szmygin, Maciej Szmygin, Mateusz Cheda, Bartosz Kłobuszewski, Anna Drelich-Zbroja, Beata Matyjaszek-Matuszek

**Affiliations:** 1 Department of Endocrinology, Diabetology and Metabolic Diseases, Medical University of Lublin, 20-093 Lublin, Poland; bmm@2com.pl; 2 Department of Interventional Radiology and Neuroradiology, Medical University of Lublin, 20-093 Lublin, Poland; mszmygin@gmail.com (M.S.); mateusz.cheda@gmail.com (M.C.); klobuszewskib@gmail.com (B.K.); zbroanna@interia.pl (A.D.-Z.)

**Keywords:** obesity, fMRI, central nervous system, reward-related brain regions, appetite

## Abstract

Obesity is becoming one of the major global health concerns. This chronic disease affects around 650 million people worldwide and is an underlying cause of a number of significant comorbidities. According to the World Health Organization (WHO) report on obesity from 2022, this disorder became the fourth leading cause of deaths in Europe. Thus, understanding the mechanisms underlying obesity is of essential importance to successfully prevent and treat this disease. The aim of this study was to review the current insights into the potential role of fMRI in discovering the mechanisms underlying obesity on the basis of recent scientific literature published up to December 2022 and searches of the PubMed, Google Scholar and Web of Science databases. The literature assessed indicated that a growing body of evidence suggests that obesity leads to changes in both structure and connectivity within the central nervous system. Emerging data from recent functional magnetic resonance imaging (fMRI) studies prove that obese individuals present an increased motivational drive to eat as well as impaired processing in reward- and control-related brain regions. Apart from this, it is clear that fMRI might be a useful tool in detection of obesity-induced changes within the central nervous system.

## 1. Introduction

Overweight and obesity are becoming one of the major current global health concerns [1,2,3,4]. These disorders are defined as abnormal, excessive fat accumulation that may have a negative effect on health [5]. Body mass index (BMI) is a commonly used screening tool that provides a simple way of determining whether a person has a healthy weight for their height. While BMI is a useful measurement for most people, it may not be accurate for everyone as it does not take into account other factors such as muscle mass, bone density, overall body composition and racial and sex differences [6]. Nonetheless, in accordance with the World Health Organization (WHO) definition, having a BMI of 18.5 to 24.9 (defined as the body mass divided by the square of the body height and expressed in units of kg/m^2^) is regarded to be a normal weight for adults, a BMI of 25 to 29.9 is considered to be overweight and a BMI over 30 allows for obesity diagnosis [6]. Obesity is then further subdivided into three categories, depending on the BMI: obesity class I with a BMI from 30 to 34.9 kg/m^2^, class II with a BMI from 35 to 39.9 kg/m^2^ and class III with a BMI above 40 kg/m^2^ [6]. Excessive weight affects around 650 million people worldwide, with the highest prevalence found in the United States (37.3%), Saudi Arabia (37.7%) and the United Arab Emirates (35.5%) [6]. It is particularly alarming that the number of obese individuals worldwide has nearly tripled in the last 40 years [5,6]. This chronic disease has been proven to be an underlying cause of a number of significant comorbidities, such as cardiovascular diseases, hypertension, hyperlipidemia, type 2 diabetes, musculoskeletal disorders, poor mental health and neurodegenerative disorders, all of which negatively affect the quality of life and survival [7,8,9,10,11]. Moreover, obesity has been associated with an increased risk of more than 13 types of cancers, including breast, colorectal, esophageal, kidney, gallbladder, uterine, pancreatic and liver cancer [12]. Breast cancer after menopause has been proven to be the most common obesity-associated cancer among women, while colorectal cancer is considered the most common obesity-associated cancer among men [12]. According to the WHO report on the obesity pandemic from 2022, excess body weight is found in almost 60% of adults and is the cause of more than 1.2 million deaths annually, making it the fourth leading cause of deaths in Europe [4]. Unfortunately, as obesity is believed to be a pathology with multiple causes, the exact mechanism of its development is yet not fully understood. Therefore, finding out the processes underlying this disorder is of essential importance to successfully prevent and treat obesity.

The use of neuroimaging techniques, especially functional magnetic resonance imaging (fMRI), in nutritional research has significantly increased in the past 2 decades [13]. Currently, fMRI is considered as the gold standard in the assessment of neuronal functions related to food intake and eating behaviors [5]. The central nervous system has recently drawn much attention in obesity research. Multiple neuroimaging studies undertaken in people with obesity have provided insight into the neurobiological mechanisms behind this disease [1,2,14]. A growing body of evidence suggests that obesity leads to changes in both structure and connectivity within the central nervous system (CNS), which may predispose people to overeating [1,3,9]. Emerging data from the latest fMRI studies prove that obese individuals present an increased motivational drive to eat as well as impaired processing in reward- and control-related brain regions [1,2,3,9].

The aim of this study was to review the current insights into the potential role of fMRI in discovering the mechanisms underlying obesity as well as identifying neural predictors and patients’ susceptibility to obesity treatment, based on the detection of obesity-induced changes within the central nervous system.

## 2. Materials and Methods

The search was conducted using PubMed, Google Scholar and Web of Science databases by two independent researchers (HSz and MSz), with the following keywords: “obesity” or “over-weight” in combination with “functional magnetic resonance imaging” or “fMRI”. Only works in the English language published from January 2016 to December 2022 including obese patients who underwent MRI examination were included.

## 3. Results and Discussion

A total of 341 full-text articles were assessed for eligibility. Case reports and case series with small sample sizes as well as articles reporting data different from those of interest were excluded (*n* = 274). Furthermore, narrative reviews (*n* = 3) and studies with incomplete data (*n* = 31) were excluded. Finally, 40 studies were included and reviewed. After careful evaluation of the papers, 4 reports older than previously intended were added due to their importance, which resulted in 44 studies included in the presented review.

### 3.1. Food Intake Regulation

The brain plays a central role in the regulation of food intake and energy homeostasis, as it integrates multiple signals from both adipose tissue and the gastrointestinal tract that influence eating behavior [13]. The balance between energy intake and expenditure is maintained through a complex interplay of nutritional, neuronal and hormonal stimuli. The hypothalamus is widely regarded as a key structure responsible for the homeostatic regulation of food consumption. However, recently it has been emphasized that appetite and body weight in humans are controlled not only by the homeostatic network, but also by other brain regions including the reward, emotion/memory, attention and cognitive control systems [1,2,9,14]. All those circuits are closely related and interact with each other to regulate eating behavior.

#### 3.1.1. Homeostatic Brain System

**Figure 1:**
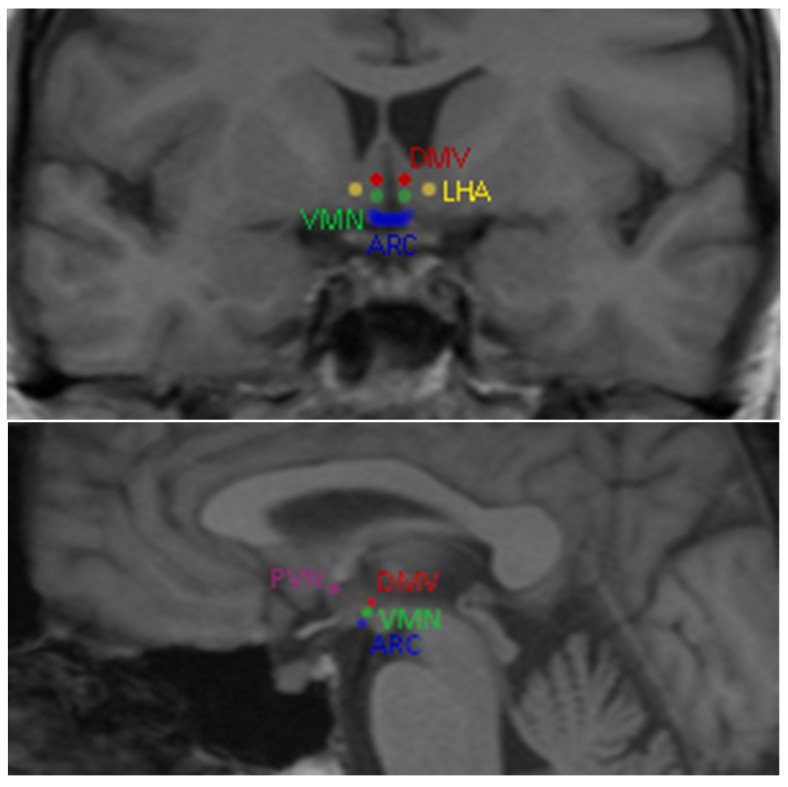
Low-calorie food vs. high-calorie food reward-related brain area activation among obese individuals. The arcuate nucleus (ARC), paraventricular nucleus (PVN), ventromedial nucleus (VMN), dorsomedial region (DMV) and lateral hypothalamic area (LHA).

Global energy balance and appetite are regulated mainly by the hypothalamic neurons [5,15,16]. Five hypothalamic nuclei have been associated with food intake regulation: the arcuate nucleus (ARC), paraventricular nucleus (PVN), ventromedial nucleus (VMN), dorsomedial region (DMV) and lateral hypothalamic area (LHA) [17] (Figure 1).

Depending on the energetic state, those regions are under the influence of orexigenic or anorexigenic peptides maintaining the body’s metabolic homeostasis [5,17]. The orexigenic peptides, such as ghrelin, neuropeptide Y (NPY) and agouti-related peptide (AgRP), stimulate the hunger-related regions of the hypothalamus in response to fasting or hypoglycemia, which promotes food intake [5]. On the other hand, the anorexigenic peptides, including cholecystokinin, YY peptide (PYY), glucagon-like peptide 1 (GLP_1), leptin and insulin influence the satiety-related region, causing appetite inhibition [5].

#### 3.1.2. Reward System

The primary cause of obesity is an energy imbalance between calories consumed and expended, which mainly results from an increased intake of energy-dense foods, high in fats and sugars and commonly regarded as highly palatable [5,6,18]. Exposure to these foods disrupts appetite regulation and promotes hedonic eating behavior, which can be defined as eating solely to produce pleasurable feelings, independently from homeostatic needs and energy status, even in the absence of physical hunger. Such a dietary pattern is mainly controlled by the reward system which is a functional unit of the limbic system [5,17,18]. This mesocorticolimbic circuit, which is responsible for reward consumption, learning, memory and addiction behaviors, is mainly composed of dopaminergic neurons located in the ventral tegmental area of the midbrain, nucleus accumbens and prefrontal cortex [5].

#### 3.1.3. Emotion/Memory Systems

Emotions, including joy, anger, fear, sadness and stress, are well-known factors influencing eating behavior. The amygdala is the main structure regulating food intake in response to emotions, and increased activation of this brain region may lead to dysfunctional eating patterns [19]. Memory, on the other hand, mainly controlled by the hippocampus, may also be responsible for abnormal food consumption [19]. It is suggested that hippocampal neurons are involved in the active maintenance of episodic memories, as well as the formation of long-term episodic memories; thus, hippocampal-dependent functions involved in regulating food intake could include declarative memory processes, as eating palatable foods can become an episodic memory that is integrated to long-term memory and recalled in the future [19]. The hypothalamus, the amygdala and the limbic system, including the hippocampus, are extensively interrelated with each other, regulating feeding and emotional behavior.

#### 3.1.4. Attention System

It has been observed that people suffering from obesity tend to pay more attention to food cues than normal-weight individuals [19]. The brain areas responsible for attention include parietal and occipital cortices as well as some areas of the frontal cortex [19]. It appears that attention-related regulation of brain activity in response to food cues and increased activity in response to food cues in parietal and visual cortices, especially in the fed state, can lead to overeating and difficulties losing weight [19].

#### 3.1.5. Cognitive Control System

Recently, attention has been drawn towards the importance of cognitive processes in the control of body weight and energy intake. The cognitive control network mainly consists of the prefrontal cortex and supervises executive functions including the inhibition of prepotent responses [19]. It allows individuals to decide when to start eating, choose the foods that they want to eat and refuse palatable foods even when they are hungry, knowing that this would not be the healthier choice [19]. Recent studies have demonstrated that individuals with obesity exhibit deficits in inhibitory cognitive control both in general and food-specific tasks [19]. Impaired inhibitory control has been proven to be associated with high-calorie food intake and resistance to losing weight [19].

#### 3.1.6. Control of Glucose Effectiveness

Control of glucose effectiveness quantifies the ability of glucose to promote its own disposal independently of any increase in insulin concentration. According to recent findings, it is emerging as an important mechanism by which the brain controls glucose homeostasis [20,21]. Evidence in the literature has shown that glucose effectiveness deteriorates in overweight individuals compared to those in the normal BMI range. In their study, Morettini et al. concluded that glucose effectiveness and its major component, i.e., glucose effectiveness at zero insulin (GEZI), deteriorate in overweight individuals compared to those in the normal BMI range, without further deterioration when BMI increases above 30 [21]. Alonge et al. introduced a model in which normal glucose homeostasis hinged on intact brain sensing of the circulating glucose level and concluded that dysfunction of this sensing process could be acquired in association with obesity and might play a central role in type 2 diabetes pathogenesis [20].

### 3.2. The Role of Brain Imaging in Obesity

Neuroimaging techniques have been widely used in recent research on the brain processes associated with the development of obesity [14]. Apart from structural imaging, functional magnetic resonance has been extensively applied to examine the differences in brain activation between obese and normal-weight individuals [14]. fMRI methods rely on the detection of stimuli-induced activation of neuronal activity and connectivity, which is associated with changes in perfusion and hemoglobin oxygenation [5,9]. Blood oxygen level-dependent (BOLD) imaging is the most widely used technique for mapping brain activity [5,9]. It is based on the detection of increases in oxygen delivery to various brain regions using the differences in the magnetic properties of deoxyhemoglobin (acting as paramagnetic) and oxyhemoglobin (acting as diamagnetic) [5,9].

Cue reactivity is one of the most frequently used paradigms incorporated in fMRI studies. Sensory cues (visual, olfactory or gustatory), acting as conditioned stimuli, are often applied to trigger central reactions [1]. The most frequently used paradigms require the presentation of food images or food delivery. The most distinct advantages of fMRI studies, namely its low invasiveness and the lack of need for gadolinium contrast administration, have made this technique one of the best diagnostic methods currently available for the examination of regional brain activations in response to specific cues or tasks [5].

### 3.3. Obesity-Induced Changes in the Brain

Obesity causes a number of complications involving multiple organs, including the neural system [9]. The latest studies suggest that obesity could be an underlying cause of the development of neurodegenerative, cognitive and dementia disorders, such as Alzheimer’s disease (AD) [5]. It is well established that excessive weight is associated with abnormal cytokine production, resulting in a low-grade systemic inflammation response, which affects, among others, the nervous tissue [5,9]. This process induces changes in cerebral microstructure, metabolism and function, causing gradual synaptic loss and cognitive decline [5,9]. Of note, it seems that those changes are reversible to some extent, as in a study by Veronese et al. on 1019 individuals, weight loss resulted in an improvement of memory and attention [5]. In addition to this, recent data confirm the presence of obesity-related systemic inflammation in the hypothalamus, amygdala and hippocampus [5]. Moreover, it has been observed that this neuroinflammation reduces the integrity of brain structures involved in reward and feeding behaviors. Neuroimaging techniques, such as fMRI, are helpful tools for closely investigating obesity-induced changes in brain function.

### 3.4. Functional Magnetic Resonance Changes in Obesity

A number of current neuroimaging studies show that in overweight and obese people, structural changes within the central nervous system (CNS) are observed, including grey matter shrinkage, reduced hippocampal volume and decreased white matter integrity [1,22]. Moreover, emerging data suggest that obesity leads to changes in brain connectivity, which may influence motivation to eat, resulting in weight gain [1]. A strong positive correlation between reductions in global brain connectivity (GBC) and increasing body mass index (BMI) has been shown in recent neuroimaging studies [1].

The differences in brain activation between obese and lean individuals in response to visual as well as olfactory cues have been widely examined in fMRI studies [1,9,14,15,23,24]. In lean individuals, oral ingestion of glucose leads to a transient decrease in brain reactivity in hypothalamic ventromedial and paraventricular nuclei [1,23,25]. Yet, this reduction is blunted in obese individuals [2,23,26]. Moreover, normal-weight people exhibit a significant decrease in reward-related response to images of palatable and high-calorie food in fasted vs. fed state in resting-state fMRI examination [7,23]. However, individuals with obesity show a significant increase in connectivity between these areas both pre- and post-meal [7,23]. Interestingly, a study by Zhang et al. demonstrated that in obese individuals, altered neural activities in several brain areas, including reward, visual, learning/memory, emotional and cognitive processing regions, persist even under non-food stimulation [3,27].

**Figure 2:**
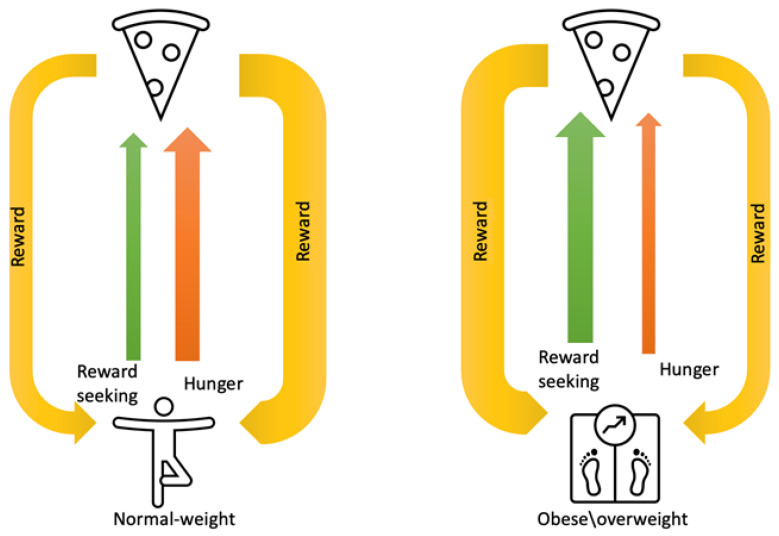
Food intake regulation in normal-weight person versus obese individual.

Food intake in obese and overweight people is more reward-based and less dependent on homeostatic needs [2,28,29] (Figure 2). It has been proved that in individuals with obesity, appetitive drive and reward perception are altered, which promotes overeating, independently of physiological satiety [2,14,15]. Additionally, people with obesity show increased activation of reward-related brain regions (such as the insula, amygdala, orbitofrontal cortex and striatum) when they view pictures of highly palatable foods, which continues even post-meal, when satiated [1,7,14,15,29]. Thus, the desire to eat becomes disconnected from hunger and satiety and persists beyond satisfying nutritional requirements, driven by hedonic responses and emotional factors [1].

**Figure 3:**
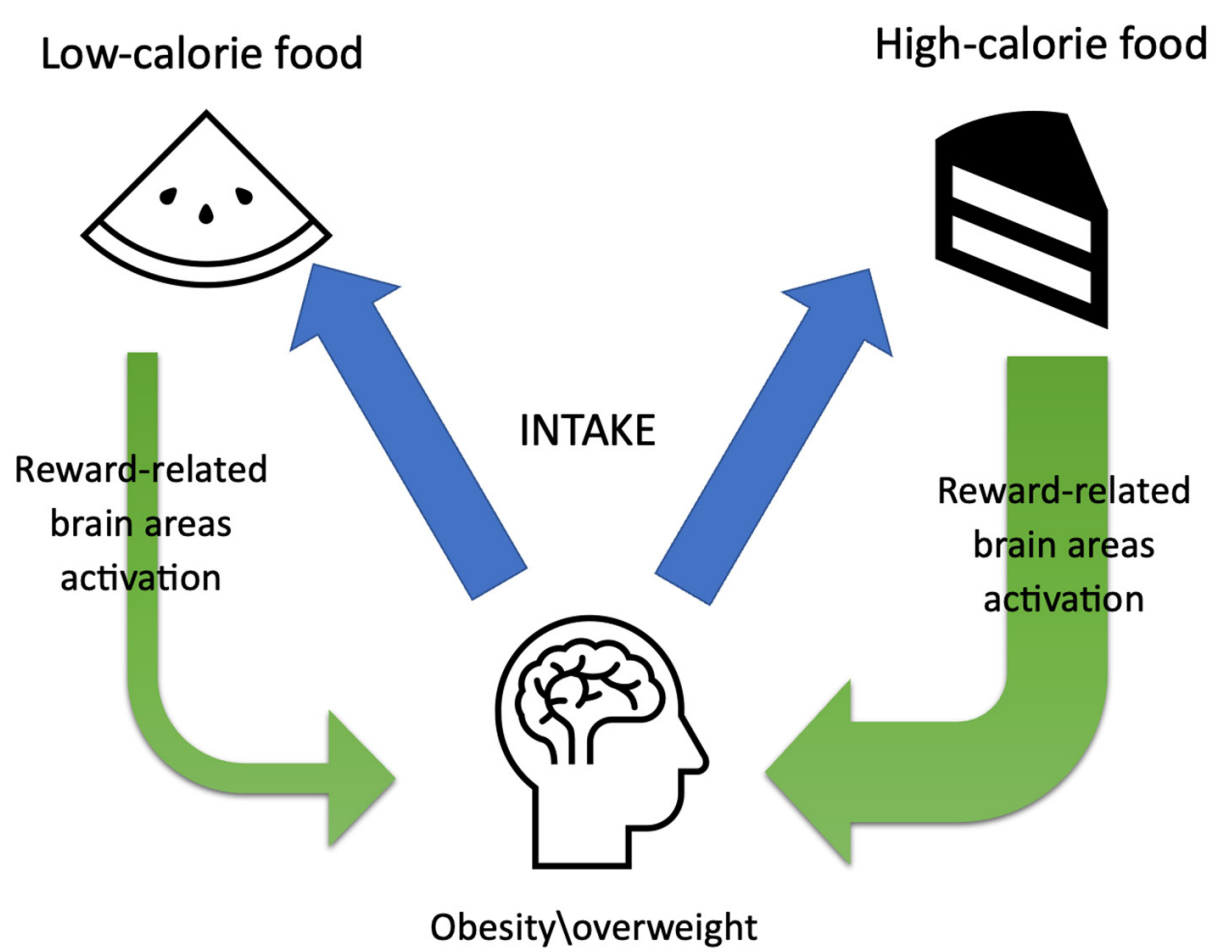
Low-calorie food versus high-calorie food reward-related brain area activation among obese individuals.

Moreover, obese individuals consider energy-dense foods as more pleasurable, and such meals induce stronger reward responses in this group of patients when compared to normal-weight individuals [1] (Figure 3). fMRI responses to high- and low-calorie foods have shown increased activation in reward-related brain regions in obese compared to lean individuals [1,7,14,30,31]. Interestingly, the degree of hyperactivation is positively correlated with increasing BMI [1,7]. These results are in line with the food addiction model of obesity, in which neurobiological alterations in the striatum result in unhealthy food intake habits [14,32].

While most studies so far have used food images as fMRI stimuli, attention has recently been drawn to chemosensory food cues such as odor and taste [33,34,35]. Han et al. examined brain activation in response to odors related to high- and low-energy-density foods in obese individuals [33]. The results showed heightened brain activation in response to chocolate (high energy density) vs. cucumber (low energy density) odors in reward-related and flavor-processing brain areas in the group with obesity when compared to individuals with normal weight [33]. These results are in line with previous studies proving obesity-related hyperactivation of reward-related brain regions in response to high-calorie food images [33].

Interestingly, it has been suggested that in people suffering from obesity, overeating may also result from impaired self-control and executive function [1,36,37]. Mildly decreased activation of frontal regions, responsible for inhibition and cognitive control, is observed in obese individuals in response to visual food cues [2]. What is more, obese people show difficulties in decision-making, planning and problem-solving as a result of reduced prefrontal cortex function, which is known as a control center for motivated behavior [1].

### 3.5. The Gut–Brain Axis

The activity of brain areas controlling energy homeostasis is also influenced by signals from the gastrointestinal tract, including gut hormones such as ghrelin, leptin, peptide tyrosine (PYY), cholecystokinin (CCK) and glucagon-like peptide 1 (GLP-1) [1,21,38,39].

Zanchi et al. reviewed recent studies analyzing the relationship between the changes in plasma concentrations of the main gut hormones and the activity of brain regions regulating food consumption [39]. According to the authors, ghrelin, which is an appetite stimulator, increases the activity of the prefrontal cortex, amygdala and insula and reduces the activation in subcortical areas including the hypothalamus [39]. On the other hand, plasma levels of satiety regulators such as leptin, PYY and GLP-1 affect the same brain regions conversely [39]. Moreover, ghrelin administration to normal-weight individuals was shown to increase neural responses to food cues in the amygdala, OFC, anterior insula and striatum [1,21,39]. Interestingly, in obese individuals, glucose administration does not suppress plasma ghrelin and fMRI activity in homeostatic and reward regions of the brain, but it suppresses neural activity in executive control regions [23]. A similar systematic review was carried out by Althubeati et al. and revealed that satiety regulators modulate the activity of brain regions such as the caudate nucleus, hypothalamus, thalamus, putamen, anterior cingulate cortex, insula and OFC, whereas appetite regulators have a greater influence on the insula, amygdala, hippocampus and OFC [38].

The effects of GLP-1 on the central nervous system have been widely examined as GLP-1 analogs are considered a therapeutic option for obesity treatment. It has been confirmed that peripheral GLP-1 can exert a direct central action and modulate neuronal activity, since GLP-1 receptors are widely expressed in several areas of the central nervous system, including the frontal, parietal, temporal and occipital cortices, basal ganglia and hypothalamus [40]. Pannacciulli et al. carried out a study to investigate whether the postprandial GLP-1 response correlates with changes in neural activity in brain regions involved in food intake regulation, by measuring the changes in regional cerebral blood flow using PET scanning after administration of a liquid formula meal to forty-two participants [40]. The results demonstrated that the postprandial GLP-1 response is associated with the activation of the dorsolateral prefrontal cortex and hypothalamus, proving that GLP-1 has an effect on brain regions involved in the regulation of eating behavior in humans [40]. The authors acknowledged the limitations of PET as an imaging technique, including spatial resolution, contrast resolution of individual subtraction images and accuracy of the image deformation. However, they provide other studies that also implemented this technique [41].

### 3.6. Weight Loss Interventions

A variety of preventative measures have been developed to manage the obesity pandemic. Dietary and lifestyle modifications constitute the first-line therapy for patients with overweight or obesity. In addition to that, anti-obesity medications, such as GLP-1 agonists and the combination of bupropion and naltrexone, can be taken to reduce appetite. Bariatric surgery, including Roux-en-Y gastric bypass (RYGB) and laparoscopic sleeve gastrectomy (LSG), offers the most successful and sustainable long-term treatment method for morbid obesity (class III). Recently, it has been observed that effective implementation of those weight loss interventions can normalize various pathophysiological processes related to obesity. Recent data suggest that fMRI studies might be used to monitor and predict the outcome of weight loss interventions, including lifestyle changes as well as pharmacological and surgical methods [8,14,42].

Herman et al. carried out a six-month prospective study to examine the association between early weight loss intervention effects on food-cue reactivity in the striatum and individuals’ BMI [14]. Participants were recommended to consume a low-calorie diet, 1500 kcal/day, for a period of six months. Food-cue reactivity in the striatum was measured at baseline and one month into the weight loss program, and the results were correlated with weight changes observed at the end of the six-month intervention. According to the authors, the change in food-cue reactivity in several regions of the striatum, including the bilateral putamen, right pallidum and left caudate, between baseline and one month in the weight loss program predicted BMI change after six months [14].

A study by van Opstal et al. aimed to investigate the influence of weight loss interventions on brain activity in obese individuals, using whole-brain resting-state functional magnetic resonance imaging [16]. It has been previously stated that in people with obesity, increased activity of brain regions responsible for energy regulation, both homeostatic and hedonic areas, is observed [16]. In this study, the authors demonstrated that an 8-week weight loss intervention (low-calorie diet), significantly decreased neural activity, especially in brain regions involved in the hedonic and executive control [16]. Interestingly, the neural activity in those regions correlated with BMI and leptin concentrations [16].

Yeung et al. performed a meta-analysis of the results from fMRI studies on the effects of anti-appetite medications on food processing within the central nervous system [42]. A significant reduction in brain activity in response to visual food cues in regions relevant to taste processing, including the right claustrum and insula, was detected in the group receiving anti-appetite medications [42].

Similar results on the effects of combined naltrexone/bupropion treatment on brain activity in obese individuals were presented by Wang et al. [43]. A noticeably decreased activation in the hypothalamus and an increased activation in the brain areas responsible for inhibitory control in response to visual food cues were observed [43].

GLP-1 analogs are a group of anti-obesity medications that act both on the homeostatic hunger–satiety brain regions and on the mesolimbic system, exerting an inhibitory effect on the reward system [44]. In a recent observational prospective study on 69 obese individuals, the GLP-1 analog semaglutide significantly ameliorated abnormal eating patterns, including emotional eating and sweet cravings, after three months of treatment [41]. Furthermore, it has been observed that GLP-1 agonist administration reduced brain hyper-responsiveness to food pictures in the insula, amygdala, putamen and OFC in obese people compared to lean individuals [25]. In a randomized, placebo-controlled, crossover study the authors observed that another GLP-1 analog, liraglutide, altered brain activity related to highly desirable food cues, decreasing activation in the insula and putamen, which are areas involved in the reward system, and acting through hypothalamic circuitry instead [45].

A growing body of evidence proves that bariatric surgery restores obesity-related structural and connectivity changes in the central nervous system and leads to neuroplastic recovery [1,8,9,10,23,46]. Recent studies show that increased food-cue responsivity observed in obese people resolves following bariatric surgery [1,46]. In a study on 26 individuals with obesity, Bach et al. proved that following bariatric surgery, the neural response to food cues decreases noticeably in reward-related brain regions and increases in areas promoting executive control [46]. Additionally, bariatric surgery changes the perception and hedonic value of food [1]. A marked change in food choice is seen following bariatric surgery as people assign lower hedonic values and reduced liking scores to tastes previously considered pleasant [1].

The most important findings presented above are shown in Table 1.

## 4. Future Directions and Perspectives

The clinical utility of fMRI is yet to be fully discovered. Future research is necessary to understand the exact mechanisms underlying excessive food intake as well as to help implement the most suitable therapy for this disorder. In addition to that, fMRI could also be useful in identifying neural predictors and patients’ susceptibility to obesity treatment, thus making it possible to implement a tailored therapy for each individual. Prospective longitudinal studies are needed to verify the predictive value of neurological markers. Interventions aimed at decreasing reward regions’ responsivity to food cues and increasing inhibitory control could be undertaken in the future to reduce weight gain and successfully treat obesity.

## 5. Conclusions

In summary, a number of recent studies indicate that fMRI might be a useful tool in the detection of obesity-induced changes within the central nervous system. However, it remains unclear whether those alterations are primary or a consequence of obesity. Additional studies are needed to further explore the relationship between eating behavior and neuronal activation as understanding of the physiological systems regulating food intake and body weight is of essential importance for effectively treating an escalating global obesity pandemic.

## Figures and Tables

**Table 1:** Summary of studies on fMRI in obese patients.

Study	No. of Participants	Methodology	fMRI Protocol	Main Findings
Stopyra et al. [2]	50 25 normal-weight and 25 obese	Water vs. glucose injected into the stomach	Event-related fMRI paradigm	− Regulation of food craving is associated with increased neural cognitive top-down control
Zhang et al. [3]	46 23 normal-weight and 23 obese	Analysis of amplitude of low-frequency fluctuation and functional connectivity	Resting-state fMRI	− Reorganized neural network presented as a bilateral cross-regulation pattern across hemispheres between reward and various appetite-related functional processing regions
Karem et al. [7]	10 All participants were obese	Intranasal oxytocin administration	Event-related fMRI paradigm	− Oxytocin attenuates the functional connectivity between the ventral tegmental area and food-motivation brain regions in response to high-calorie visual food images
Bach et al. [8]	11 All participants were obese	Longitudinal reliability of neural food-cue-induced brain activation and subjective food craving ratings before and after bariatric surgery	Event-related fMRI paradigm	− fMRI might be suitable for monitoring and predicting treatment outcomes in participants with obesity undergoing bariatric surgery
Zeighami et al. [10]	57 All participants were obese	Assessing spontaneous neural activity before and after bariatric surgery	Resting-state fMRI	− Bariatric surgery-induced weight loss is associated with widespread global and regional increases in neural activity
Hermann et al. [14]	68 All participants were obese	fMRI examinations during the six-month-long intervention period with a low-calorie diet	Event-related fMRI paradigm and resting-state fMRI	− Correlation between BMI change after six months and early alterations of fMRI food-cue reactivity in the striatum
Sewaybricker et al. [15]	84 Monozygotic twins 54 Dizygotic twins 30	To test if appetitive drive varies in direct proportion to the level of body adiposity after accounting for genetic factors that contribute to both brain response and obesity risk	Event-related fMRI paradigm	− Level of adiposity was not associated with excess appetitive drive as assessed by behavioral, hormonal or fMRI measures
Simon et al. [26]	76 24 with anorexia nervosa 28 normal-weight 24 obese	Investigation of the responsivity of the hypothalamus after intragastric infusion of glucose and water	Resting-state fMRI	− Blunted hypothalamic glucose reactivity might be related to the pathophysiology of anorexia nervosa
Filbey et al. [31]	26 All participants were obese	Assessment of hyper-responsivity to reward in individuals exhibiting binge-eating behavior	Event-related fMRI paradigm	− High-calorie taste cues elicited a response in the reward system, and this hyper-responsivity increases with the number of binge-eating symptoms
Bragulat et al. [34]	10 participants 5 normal-weight and 5 obese	After 24 h of food deprivation, all individuals underwent fMRI examination while smelling four food-related odors	Event-related fMRI paradigm	− Food odors are highly naturalistic stimuli and may be effective probes of reward-related networks in the context of hunger and obesity
Wang et al. [43]	36 16 after treatment with naltrexone and SR bupropion (NB) 20 placebo	Evaluation of the effects of naltrexone and SR bupropion on functional connectivity (FC) density	Resting-state fMRI	− NB decreased local and global FC in the superior parietal cortex and decreased local FC in the medial prefrontal cortex (craving)
Farr et al. [45]	22 All participants were obese and had diabetes	To investigate if GLP-1 receptors are expressed in human brains and whether liraglutide administration affects neural responses to food cues in diabetic individuals	Event-related fMRI paradigm	− The presence of GLP-1 receptors in human brains was demonstrated − Liraglutide alters brain activity related to highly desirable food cues

## Data Availability

The data of the current study (not included in this published article) are available from the corresponding author upon reasonable request.
